# Ribosome Subunit Stapling for Orthogonal Translation in *E.* *coli*

**DOI:** 10.1002/anie.201506311

**Published:** 2015-08-26

**Authors:** Stephen D Fried, Wolfgang H Schmied, Chayasith Uttamapinant, Jason W Chin

**Affiliations:** Medical Research Council Laboratory of Molecular Biology Francis Crick Avenue, Cambridge CB2 0QH (UK)

**Keywords:** genetic code reprogramming, orthogonal ribosomes, ribosomal RNA engineering, synthetic biology, translation

## Abstract

The creation of orthogonal large and small ribosomal subunits, which interact with each other but not with endogenous ribosomal subunits, would extend our capacity to create new functions in the ribosome by making the large subunit evolvable. To this end, we rationally designed a ribosomal RNA that covalently links the ribosome subunits via an RNA staple. The stapled ribosome is directed to an orthogonal mRNA, allowing the introduction of mutations into the large subunit that reduce orthogonal translation, but have minimal effects on cell growth. Our approach provides a promising route towards orthogonal subunit association, which may enable the evolution of key functional centers in the large subunit, including the peptidyl-transferase center, for unnatural polymer synthesis in cells.

The ribosome is a large molecular machine, universally composed of two subunits, that decodes non-overlapping triplet codons in mRNAs for the encoded polymerization of amino acids into proteins.^1^ The small subunit, containing 16S rRNA, binds mRNA and decodes the interaction between codons on mRNAs and their cognate tRNA anticodons, and the large subunit, containing 23S rRNA, facilitates many functions, including peptide bond formation. While natural translation encodes the polymerization of the canonical 20 amino acids, extensions of translation for the polymerization of unnatural building blocks will unlock routes to encode and evolve new classes of polymers. However, because the ribosome is essential for proteome synthesis and many mutations in the ribosome are dominant-negative or lethal in the cell,^2^ it is challenging to alter and evolve the natural ribosome for unnatural polymer synthesis in cells.

To address the challenge of creating an evolvable ribosome, we have previously created orthogonal (O)-ribosome-O-mRNA pairs (Figure 1) in *E.* *coli*.^3^ The O-ribosome contains a mutated anti-Shine–Dalgarno (ASD) sequence within its O-16S rRNA, enabling O-ribosomes to selectively and efficiently translate O-mRNAs bearing the orthogonal Shine–Dalgarno (O-SD) sequences. Likewise, O-mRNAs are not translated by endogenous ribosomes. Because the orthogonal ribosome, unlike the natural ribosome, is not responsible for synthesizing the proteome, its O-16S rRNA may be evolved to perform new functions. We have previously evolved ribo-X in which the decoding center, within the O-16S rRNA of the orthogonal ribosome, no longer recognizes release factor 1, thereby enabling efficient incorporation of unnatural amino acids in response to the amber stop codon.^4^ We have also evolved ribo-Q, which uses extended anticodon tRNAs to efficiently incorporate unnatural amino acids in response to diverse quadruplet codons, enabling the site-specific incorporation of multiple distinct unnatural amino acids into recombinant proteins.^5^

**Figure 1:**
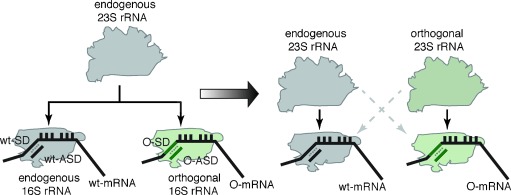
An orthogonal 16S (O-16S) rRNA contains an orthogonal anti-Shine–Dalgarno (O-ASD) sequence, which confers specificity to the small subunits that contain an O-16S rRNA to translate orthogonal mRNAs (O-mRNA) that bear orthogonal Shine–Dalgarno (O-SD) sequences. With no further components added to the cell, this O-16S rRNA shares endogenous 23S rRNA with endogenous 16S rRNA (left). Creating an orthogonal 23S rRNA that specifically functions with an orthogonal 16S rRNA (that does not function with endogenous 23S rRNA), will enable an altered 23S rRNA to be insulated from cellular translation and selectively used in orthogonal translation (right).

Many key ribosomal functions, including interactions with tRNAs and elongation factors, peptide bond formation in the peptidyl-transferase center (PTC), and the folding and release of the nascent chain through the exit tunnel,^1^, ^6^ are mediated by 23S rRNA within the large subunit. These functional centers cannot be evolved in the current orthogonal ribosome that uses the endogenous pool of large subunits, containing 23S rRNA, in combination with the orthogonal small subunit, containing O-16S rRNA, to translate the O-mRNA (Figure 1). Creating an O-23S rRNA that assembles into an orthogonal large subunit and is specifically coupled to the orthogonal small subunit, containing O-16S rRNA, will enable the creation of orthogonal ribosomes in which both subunits are selectively recruited to an orthogonal message (Figure 1). This will facilitate alteration and evolution of functional centers in the O-23S rRNA not possible on the endogenous 23S rRNA.

The large and small ribosomal subunits interact through non-covalent RNA–RNA interactions between 16S rRNA and 23S rRNA that bury approximately 6000 Å^2^, and these interactions are dynamically regulated through the translation cycle.^7^ Efforts to control non-covalent subunit interactions through rRNA mutagenesis have proved unsuccessful thus far. Here we investigate the creation of an orthogonal ribosome in which the O-16S rRNA is covalently attached to a 23S rRNA to create a fused rRNA (Figure 2 A). The fused rRNA assembles into a new orthogonal ribosome that translates an orthogonal message and permits mutagenesis of the 23S rRNA.

**Figure 2:**
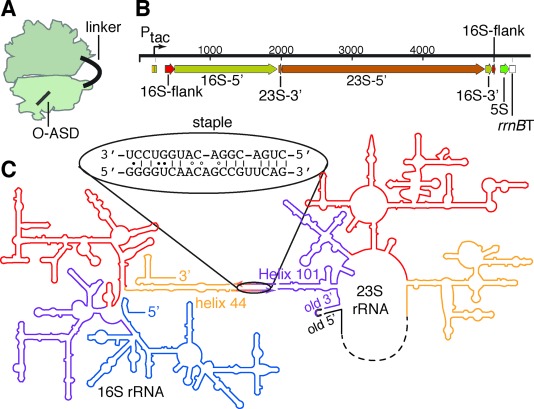
Ribosome subunit stapling. A) Illustration of the stapled ribosome. B) 1-D representation of the designed rDNA operon. The 16S gene is separated into two halves (16S-5′ and 16S-3′) by the insertion of 23S rDNA. The 23S gene is circularly permuted such that a 3′-terminal segment (23S-3′) precedes its 5′-terminal segment (23S-5′). This transcript (ca. 4500 nt) is flanked by the native 16S processing sites (flanks). The 23S processing sites (which would normally liberate the 23S rRNA into a separate RNA molecule) have been deleted. C) 2-D representation of the stapled ribosome, illustrating the linker sequence used to staple helix 44 (h44) of 16S rRNA to Helix 101 (H101) of 23S rRNA into a single rRNA molecule.

We envisioned joining the two subunits by reorganizing the *rrnB* operon such that a 23S rRNA would be nested within the 16S rRNA as a large insertion (Figure 2 B). We were encouraged by previous observations that in various organisms 16S rRNAs can exist in multiple fragments or with long insertions.^8^ Moreover, the 23S rRNA is tolerant to circular permutation,^9^ indicating that it might be possible to circularly permute the 23S rRNA to open up new 5′ and 3′ termini at positions proximate to surface exposed features of the 16S rRNA, and then insert this permuted 23S at that site on the 16S, connected on both ends by an RNA linker (Figure 2 C).

We used high-resolution structures of *E.* *coli* ribosomes7a, 10 and phylogenetic variation^11^ in rRNA sequence to identify regions of 23S rRNA and 16S rRNA that come close in space, and may be tolerant to insertion (Supporting Information, Figure S1 A). This analysis identified Helix 101 (H101) on the 23S and helix 44 (h44) on the 16S as an excellent pair of sites to test our strategy (Figure S1 B). These helices come into close contact (3 nm) in ribosome structures,7a, 10 and are tolerant to insertions as judged by their natural phylogenetic variation11b and previous genetic engineering.8b Moreover, these helices are distal from the corridor through which tRNAs transit and elongation factors dock (Figure S1 B). Taking a rational structure-based approach, we opted to circularly permute 23S at H101 and insert it within 16S, at the terminal loop of h44 (Figure 2 C). We linked the 16S and 23S sequences via the J5/J5a region from the *Tetrahymena* group I self-splicing intron (Figure 2 C), an RNA hinge that can toggle between an extended and “U-turning” form.^12^ This “stapled” ribosome rDNA was synthesized by overlap extension PCR (Figure S2, Table S1), cloned into a pRSF plasmid following an inducible P_tac_ promoter, and given an orthogonal ASD (O-ASD) via site-directed mutagenesis.^3^ We refer to the resulting construct as pRSF-O-ribo(h44H101).

Because the unusual topology of the O-ribo(h44H101) rRNA could complicate ribosome folding and assembly pathways,^13^ it was critical to ascertain the extent to which pRSF-O-ribo(h44H101) produces a full-length rRNA that persists in vivo. To address this question we probed RNA extracted from *E.* *coli* expressing O-ribo(h44H101) by northern blot using a biotinylated probe specific to the O-ASD sequence of the orthogonal ribosome (Figure 3 A). We detected a single band at 4500 nt, demonstrating that the major species bearing an O-ASD, in cells transformed with pRSF-O-ribo(h44H101), is the full length O-ribo(h44H101) rRNA. These data suggest that translation of O-mRNAs in cells bearing pRSF-O-ribo(h44H101) results from the activity of the stapled ribosome. In control experiments, RNA extracted from cells expressing the orthogonal ribosome from pRSF-O-Ribo (a plasmid with the same copy number, encoding orthogonal ribosomes under the same promoter, but with wild-type operon topology) was probed in a northern blot with the O-ASD-specific probe. In this experiment we detected a band at 1500 nt, as expected for the 16S rRNA (Figure 3 A), and the intensity of this band was approximately four times that of the band detected for O-ribo(h44H101) rRNA (Figure 3 B). These data suggest that either the O-ribo(h44H101) rRNA is not transcribed as efficiently as the *rrnB* operon with native topology and/or a fraction of the transcript does not assemble correctly and is ultimately degraded.

**Figure 3:**
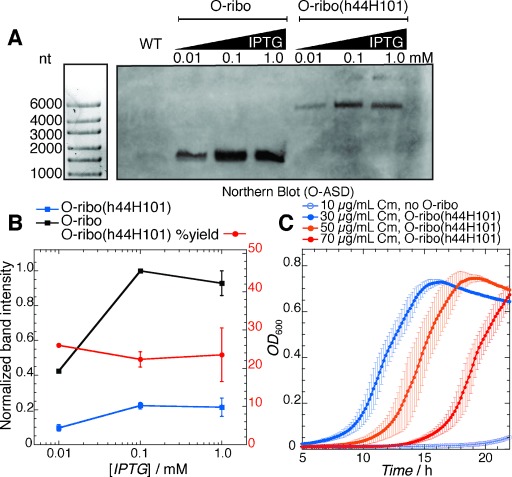
rRNA derived from pRSF-O-ribo(h44H101) is assembled into functional ribosomes in vivo. A) Northern blot using a probe specific to the O-ASD sequence detects RNAs from total RNA extracts that bear O-ASDs. Wild-type (WT) *E.* *coli*, which does not possess orthogonal ribosomes, generates no band. O-ribo possesses an O-ASD on the 16S rRNA, and generates a band at 1500 nt (the length of 16S rRNA). pRSF-O-ribo(h44H101) generates an O-ASD-containing band near 4500 nt (nucleotides). B) Increasing isopropyl β-d-thiogalactopyranoside (IPTG) concentrations up to 0.1 mm increases the expression of orthogonal ribosomes (blue and black traces). Stable O-ribo(h44H101) rRNA is generated to about 25 % the levels of O-ribo (red trace). C) Growth curves of *E.* *coli* bearing an O-cat reporter, with or without O-ribo(h44H101), at 37 °C in liquid LB media supplemented with IPTG and chloramphenicol (Cm).

To test the activity of O-ribo(h44H101) in protein translation we co-transformed pRSF O-ribo(h44H101) and an O-cat reporter in which a chloramphenicol acetyltransferase gene (cat) is downstream of an O-SD site for ribosome binding.^3^, ^4^ Following induction of rRNA synthesis with IPTG, we followed the growth of cells in different concentrations of chloramphenicol (Cm) to assess the activity of O-ribo(h44H101).

Cells bearing the O-cat reporter alone, or provided with pRSF-O-ribo but not induced with IPTG, do not grow on 10 μg mL^−1^ Cm (Figure 3 C; Supporting Information, Figure S3, Tables S2, S3). In contrast, when cells are provided with pRSF-O-ribo(h44H101) and O-cat they grow robustly on Cm concentrations up to 70 μg mL^−1^ (Figure 3 C, Table S4), indicating that pRSF-O-ribo(h44H101) directs the synthesis of ribosomes that specifically translate the orthogonal message. The activity of O-ribo(h44H101) in the assay is lower than that of O-ribosomes with independent subunits produced from a standard operon, which confer Cm resistance up to 200 μg mL^−1^, but not 300 μg mL^−1^, in our assay (Figure S4, Table S5). We further demonstrated the activity of O-ribo(h44H101) in an independent assay by measuring its ability to translate O-luciferase (a luciferase gene expressed from an orthogonal ribosome binding site),^4^ as measured by a luciferase activity assay (Figure S5). This led to results that are quantitatively consistent with our observations in the chloramphenicol resistance assay.

To investigate whether the activity of the O-ribo(h44H101) is dependent on the stapled 23S rRNA (Figure 4A, B) we introduced two mutations (G2252A and G2553C) into the 23S portion of O-ribo(h44H101), creating O-ribo(h44H101(G2252A)) and O-ribo(h44H101(G2553C)). The guanosines targeted for mutation base pair with the universally-conserved 3′-CCA ends of tRNAs and their mutation is reported to severely hinder protein synthesis.^14^ When O-ribo(h44H101(G2252A)) and O-ribo(h44H101(G2553C)) were co-transformed with O-cat, cells failed to grow on 30 μg mL^−1^ Cm after 20 h (Figure 4 A; Supporting Information, Tables S6, S7), while O-ribo(h44H101) grew robustly on 30 μg mL^−1^ Cm (Figure 3, Figure 4 A) and continued to survive on Cm concentrations up to 70 μg mL^−1^ (Figure 3 C). These data are consistent with the un-mutated large subunit of O-ribo(h44H101) being functional and important in orthogonal translation.

**Figure 4:**
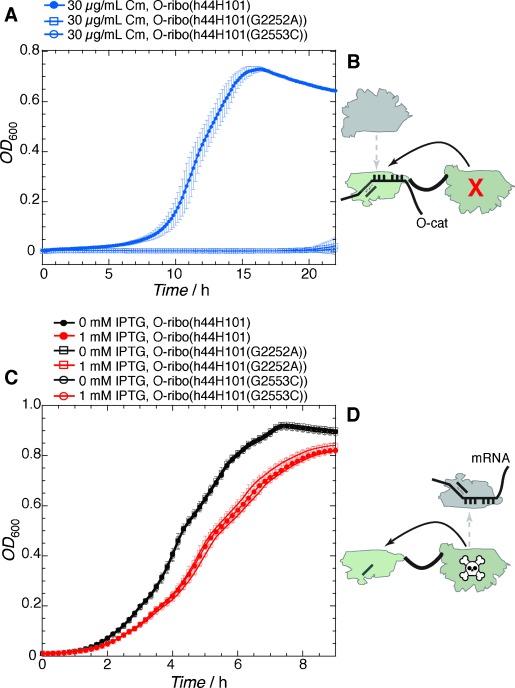
Investigating the orthogonality of the small and large subunit portions of O-ribo(h44H101) with respect to endogenous ribosome subunits in translation. A) Growth curves of *E.* *coli* bearing an O-cat reporter and O-ribo(h44H101), with or without mutations that inactivate the large subunit portions for translation, at 37 °C in liquid LB media supplemented with IPTG and Cm. Inactivating the stapled large subunit results in a decrease in Cm resistance. B) For orthogonally associating ribosome subunits, the small subunit portion of O-ribo(h44H101), after forming an initiation complex with an O-cat mRNA, would be primarily captured by the stapled large subunit (solid arrow) and not the endogenous large subunit (dashed arrow). C) Growth curves of *E.* *coli* bearing O-ribo(h44H101), with or without mutations in the large subunit portion that confer a dominant-lethal phenotype in cells, at 37 °C in liquid LB. Red curves +IPTG; black curves −IPTG. D) For orthogonally associating ribosome subunits, dominant-negative mutations in the large subunit portion would not substantially affect cell fitness, and endogenous small subunit-mRNA complexes would minimally capture the large subunit (dashed arrow).

Although G2252A and G2553C in 23S rRNA are reported to be dominant-negative when expressed in cells,^14^ they were readily introduced into O-ribo(h44H101) by site-directed mutagenesis. Moreover, the reduction in growth imposed by these mutant ribosomes (O-ribo(h44H101(G2252A)) and O-ribo(h44H101(G2553C))), with respect to O-ribo(h44H101), was small, even with maximum IPTG induction of rRNA expression (Figure 4 C, Supporting Information, Tables S8–S10).

These data indicate that the mutations do not have a substantial dominant-negative effect on cellular translation in the stapled ribosome, consistent with the large subunit of O-ribo(h44H101) being functionally insulated from the endogenous small subunit (Figure 4 D).

In conclusion, we have described the rational, structure-based design of a stapled orthogonal ribosome. Our design inserts a circularly permuted 23S rDNA into the 16S rDNA at sites determined by structural and phylogenetic analysis, and uses an RNA hinge to staple the two subunits and facilitate subunit association and disassembly. Our results indicate that the stapled orthogonal ribosome allows the effects of mutations in 23S rRNA to be specifically coupled to translation of an orthogonal message and insulated from endogenous translation. Future work will focus on optimizing the activity of our rationally designed stapled ribosome, and fully characterizing the extent to which orthogonality in subunit association (Figure 1) may be achieved through the stapling of ribosome subunits. We anticipate that the development of stapled orthogonal ribosomes may further extend orthogonal translation, and enable further progress on the genetically encoded synthesis of unnatural polymers in cells.

## Supporting Information

As a service to our authors and readers, this journal provides supporting information supplied by the authors. Such materials are peer reviewed and may be re-organized for online delivery, but are not copy-edited or typeset. Technical support issues arising from supporting information (other than missing files) should be addressed to the authors.

miscellaneous_information
