# Supplementary material for: Ribosome Subunit Stapling for Orthogonal Translation in E. coli
Source: Angew Chem Int Ed Engl. 2015 Aug 26;54(43):12791–4. doi: 10.1002/anie.201506311 (PMC4678508; doi:10.1002/anie.201506311)
Supplement: Supplementary file 1 [file anie0054-12791-sd1.pdf]

Angewandte Chemie
Eine Zeitschrift der Gesellschaft Deutscher Chemiker

Supporting Information

Ribosome Subunit Stapling for Orthogonal Translation in *E. coli*

*Stephen D. Fried, Wolfgang H. Schmied, Chayasith Uttamapinant, and Jason W. Chin**

anie_201506311_sm_miscellaneous_information.pdf

I. Supplementary Methods

1. Molecular Biology Methods

General. All oligonucleotides used for cloning were purchased from Sigma-Aldrich with desalting and no further purification. All restrictions enzymes and ligases were purchased from New England Biolabs and were used with the buffers and protocols provided by the manufacturer. In general, PCR reactions were carried out on 25 μL scales using the 2x Q5 polymerase master mix (New England Biolabs) according to the manufacturer's instructions (extension at 72°C for 0.5 min/kbp), unless otherwise stated. Annealing temperatures were set based on the melting temperatures assigned by SnapGene 2.7.3 (T_m ± 3). Gibson assembly was carried out using the 2x Gibson master mix (New England Biolabs) according to the manufacturer's instructions. DNA purifications following PCRs, restriction digestions, and separations by agarose gel electrophoresis were carried out with silica-gel columns (Qiagen) according to the manufacturer's protocols. For most applications, plasmid transformations was carried out by electroporating 50 μL of electrompetent DH10B cells with 1 μL plasmid at 2420 V for 5 ms in electrocuvettes with 2 mm gaps. Site-directed mutagenesis was carried out with the Q5 mutagenesis kit (New England Biolabs), which calls for transformation via heat shock of chemically competent NEB 5-alpha cells. For cloning applications, transformants were plated on LB agar supplemented with the appropriate antibiotics (50 μg/mL kanamycin for pRSF, 15 μg/mL tetracycline for p15A). Generally, 4–6 single colonies were selected, inoculated in selective media, and grown overnight. Plasmids were purified and extracted from cell cultures grown in LB media supplemented with antibiotics at working concentrations. Plasmid DNA was purified using the spin mini-prep kit or the midi plus kit (Qiagen), and always analyzed by Sanger sequencing (GATC Biotech) after each step.

Construction of pRSF- O-ribo(h44H101). A fragment containing the pRSF- O-ribo(h44H101) insert (which begins and ends with the 16S flanking

sites) was generated by overlap extension PCR from four different fragments, each of which was generated from four separate PCR reactions using pKK3535 (a vector that encodes the rrnB operon; see http://cgsc.biology.yale.edu/Site.php?ID=122424) as a template. To prepare the 16S-5' fragment, template was mixed with 16Sstart-f and j1-16S1449-r2 (see Table S1). To prepare the 23S-3' fragment, j1-23S2861-f2 and 23S3'-r were mixed without template (the two primers self-anneal to generate the 85 bp product). To prepare the 23S-5' fragment, template was mixed with 23S5'-f and j1-23S2856-r2. To prepare the 16S-3' fragment, template was mixed with j1-16S1454-f2 and 16Send-r. The four fragments were restricted with DpnI, column purified, and then 0.16 pmol of each was added to a new PCR reaction using an annealing temperature of 66°C, an extension time of 3 min, and 16 cycles (assembly step). Finally, 1 μL of the reaction medium from the assembly step was introduced to a new 40 μL-scale PCR reaction, treated with 16Sstart-f and 16Send-r as primers, and cycled 25 times with an annealing temperature of 68°C and an extension time of 3 min (purifying step). The PCR product was cloned into the pTOPO vector using the Zero Blunt TOPO PCR cloning kit (Life Technologies) according to the manufacturer's instructions. Colony PCRs (using 16Sstart-f and 16Send-r as primers) were used to determine which of the resultant clones contained the long ~5000 bp insert, of which half did. Positive clones were grown overnight in selective media, and pTOPO- ribo(h44H101) was isolated from the resulting cultures. h44H101 was next cloned into the pRSF backbone (which contains the inducible P_{tac} promoter upstream of the rrnB operon, the lacI gene, a KanR resistance marker, and an RSF origin) used in prior studies of the orthogonal ribosome.[1,2] The linearized pRSF backbone was obtained by amplifying pRSF- O-Ribo with clone-pRSFbb-r and clone-pRSFbb-f; ribo(h44H101) was lifted from pTOPO by amplifying with 16Sstart-f and 16Send-r; the two reactions were restricted with DpnI, gel extracted and column purified, and subject to Gibson assembly to generate pRSF- ribo(h44H101). Two rounds of site-directed mutagenesis were carried out to install the orthogonal mRNA-

binding site on the 16S rDNA using 16S-ortho1-f and 16S-ortho1-r in the first round, and 16S-ortho2-f and 16S-ortho2-r in the second round.

Construction of other pRSF plasmids. Where a "wild-type" orthogonal ribosome is used in this study (i.e., a ribosome with an orthogonal anti-Shine-Dalgarno site but otherwise identical to the *E. coli* ribosome) we have used the ribosome described in Wang et al.[2] (referred to as ribo-X in the previous reference). To insert toxic mutations G2252A and G2553C into pRSF- O-ribo(h44H101), the QuickChange procedure was carried out by performing enzyme inverse PCR on pRSF- O-ribo(h44H101) with either WS160f-G2252A and WS160r-G2252A *or* WS159f-G2553C and WS159r-G2553C as primers. The PCR products were restricted with DpnI, and transformed by heat-shock into chemically competent NEB 5alpha cells. Six to ten clones of each were grown overnight in selective media, from which DNA was purified and sequenced. In the pRSF- O-ribo(h44H101) background, these mutations were successfully incorporated about one in four times; we were not able to successfully incorporate these mutations into the pRSF- O-ribo background (all clones sequenced were wild-type).

Reporter Plasmids. The primary reporter used in this study was p15A-O-cat, a plasmid containing a TetR resistance marker, a p15A origin, and an insert encoding chloramphenicol acetyl transferase with the following structure: a constitutive trp promoter, followed by an orthogonal Shine-Dalgarno site (ATATCCT) 6 bp upstream of a synthetic *E. coli* codon-optimized chloramphenicol acetyltransferase open-reading frame. This plasmid was described previously.[1]

2. RNA Methods

Total RNA extraction. Electrocompetent DH10b cells were transformed with pRSF- O-ribo or pRSF- O-ribo(h44H101) and plated on LB Agar supplemented with 50 μg mL^{-1} Kanamycin (LB-K). Colonies were saturated overnight in 5 mL cultures of LB-K. The following day, overnight cultures were

diluted to a starting OD_{600} of 0.01 and inoculated into flasks containing 50 mL LB supplemented with 50 μg mL^{-1} Kanamycin and varying concentrations of IPTG (0.01 mM, 0.1 mM, 1 mM) (LB-K{I}). Ribosomes were induced for ~4 h, until the cell cultures had an OD_{600} of 0.3–0.5. At this point, cell cultures were incubated on ice and cell pellets were harvested by centrifugation for 10 min at 4000 rpm, 4˚C. 1 mL of Trizol reagent (QIAzol, from Qiagen) was added to each cell pellet. After incubating for 5 min, 0.2 mL of cold chloroform was added, and the suspensions were mixed by shaking vigorously for 15 s. After incubating for 2 min, the suspensions were centrifuged for 15 min at 12000 g, 4˚C. 400 μL of the upper colorless phases were transferred to new tubes and precipitated by addition of 500 μL ice-cold isopropanol. After incubating for 10 min, the RNA precipitate was harvested by centrifugation for 10 min at 15000 g, 4˚C. Isopropanol was removed, and RNA was washed once with 500 μL 80% ethanol. Ethanol was removed by air drying at 37˚C for 10 min, and the RNA was resolubilized in 30–50 μL of RNase-free water. Concentrations were determined by Nano Drop to be around 2000 ng μL^{-1}.

Electrophoresis. 15 μg of RNA was mixed with 15 μL of glyoxal loading dye from the NorthernMax-Gly kit (Ambion), and incubated for 30 min at 50˚C with agitation. Agarose gels consisting of 0.6 g agarose, 6 mL 10x Gel Pre/Running buffer, and 54 mL DEPC-treated water were cast in a clean RNase-Zapped (Ambion) electrophoresis chamber. Denatured RNA was loaded into the gel and electrophoresed at 60 V for 60–80 min, till the bromophenol blue band was about two-thirds the way to the end of the gel. Gels were imaged on a ChemiDoc (Biorad) by ethidium bromide staining, and band intensities were quantitated using Image Lab (Biorad).

Northern Blotting. RNA was transferred from agarose gels to a nylon membrane using iBlot DNA transfer stacks (Life Technologies) according to the manufacturer's instructions, and hybridized to the nylon membrane by UV irradiation in a UV oven.[3] The membrane was rolled into a 50 mL falcon tube, to which 10 mL hybridization buffer (Ambion), and incubated at 37˚C for 30 min in a

spinning hybridization oven. Biotinylated probe specific to the orthogonal anti-Shine-Dalgarno (10 pM; Sigma) was added and hybridized over night. The following day, the membrane was washed twice with low stringency wash solution (Ambion): once for 10 min at room temperature, and then 5 min at 37˚C. Labeling was carried out with the chemiluminescent nucleic acid detection module (Thermo). The membrane was blocked for 15 min with blocking buffer, labeled with 16 mL blocking buffer supplemented with 50 μL streptavidin-HRP conjugate (Thermo) for 15 min, washed four times with wash buffer for 5 min apiece, and equilibrated with substrate equilibration buffer. The membrane was developed by standing for 5 min with a 1:1 mixture of stable peroxide solution and luminol solution (Thermo), and then imaged on a ChemiDoc (Biorad) in Chemi mode, using exposure times of 1–3 s.

3. Activity Assays

IPTG Growth Assays. Electrocompetent DH10b cells were transformed with various pRSF plasmids encoding different (orthogonal) ribosomes, and plated on LB Agar supplemented with 50 μg mL^{-1} Kanamycin (LB-K). Colonies were inoculated into 5 mL LB-K and saturated overnight. The overnight culture was diluted to a starting OD_{600} of 0.02 into a new 5 mL culture consisting of LB supplemented with 25 μg mL^{-1} Kanamycin (LB-K/2) and incubated for 4 h. 4 μL of this culture was transferred to wells in a 96-well plate containing 200 μL of LB-K/2 media further supplemented with various concentrations of IPTG (LB-K/2,{I}). Each experiment was conducted in triplicate by dispensing 4 μL of recovery into three separate but identically prepared wells. Plates were sealed with parafilm to prevent evaporation and loaded into a Tecan plate-reader equilibrated to 37˚C. Cells were agitated at 37˚C, and absorbance at 600 nm was recorded for each well every 10 minutes for ca. 24 h.

Chloramphenicol Growth Assays. Electrocompetent DH10b cells were co-transformed with the reporter construct p15A-oCAT and various pRSF plasmids encoding different orthogonal ribosomes, and plated on LB Agar

supplemented with 15 μg mL^{-1} Tetracycline and 50 μg mL^{-1} Kanamycin (LB-TK). Colonies were inoculated into 5 mL LB-KT and saturated overnight. The overnight culture was diluted to a starting OD_{600} of 0.02 into a new 5 mL culture consisting of LB supplemented with 7.5 μg mL^{-1} Tetracycline, 25 μg mL^{-1} Kanamycin, and typically 0.1 mM IPTG (LB-T/2,K/2,I) and incubated for 4 h. 4 μL of this culture was transferred to wells in a 96-well plate containing 200 μL of LB-T/2,K/2,I media further supplemented with various concentrations of chloramphenicol (LB-T/2,K/2,I,{Cm}). For each experiment, a biological triplicate was generated by dispensing 4 μL of recovery into three separate but identically prepared wells. Plates were sealed with parafilm to prevent evaporation and loaded into a Tecan plate-reader equilibrated to 37˚C. Cells were agitated at 37˚C, and absorbance at 600 nm was recorded for each well every 10 minutes for ca. 24 h.

Data Analysis. Growth curves were analyzed by manually baselining the OD_{600} time series and then fitting to splines using the grofit R-package. For each time series, maximum rate, lag time, and maximum OD_{600} was extracted and data presented in the supplementary tables are means ± standard deviations for three biological replicates.

4. Luciferase Reporter Assays.

Electrocompetent DH10b cells were co-transformed with the reporter construct pR22-oDLR[2] (encoding a fusion protein between firefly and *renilla* luciferases preceded by an orthogonal ribosome binding site) and various pRSF plasmids encoding different (orthogonal) ribosomes, and plated on LB Agar supplemented with 100 μg mL^{-1} Ampicillin and 50 μg mL^{-1} Kanamycin (LB-AK). Three single colonies were inoculated into 5 mL LB-AK and saturated overnight. The overnight culture was diluted to a starting OD_{600} of 0.02 into a new 5 mL culture consisting of LB supplemented with 50 μg mL^{-1} Ampicillin, 25 μg mL^{-1} Kanamycin, and typically 0.1 mM IPTG (LB-A/2,K/2,I) and incubated for 8 h. After this growth, final OD_{600} values were recorded, and cells from 1 mL of culture

was harvested by centrifugation for 5 min at 4000 rcf at 4˚C. The cells were washed once with 1 mL PBS, and then resuspended in 150 μL of passive lysis buffer (Dual Luciferase Reporter Assay System (Promega)), in which they were lysed by two freeze-thaw cycles passing between liquid nitrogen and ice water. The lysates were clarified by centrifugation for 15 min at 20,000 rcf at 4˚C, after which 100 μL of supernatant was transferred to a fresh tube and additional passive lysis buffer was supplemented to normalize the volumes to the final OD_{600}. 10 μL of sample were transferred into an opaque 96-well plate and luminescence from *renilla* luciferase was recorded on a PheraStar (BMG LabTech) plate reader, according to the manufacturer's protocol. Data was processed in MARS. To baseline the data, we subtracted the signal from a sample comprising of pRSF-Ribo (a ribosome containing a canonical anti-Shine-Dalgarno) and pR22-oDLR (a reporter expressing orthogonal dual luciferase mRNA). Values and error bars report means and standard deviations from biological triplicates.

II. Supplementary Figures

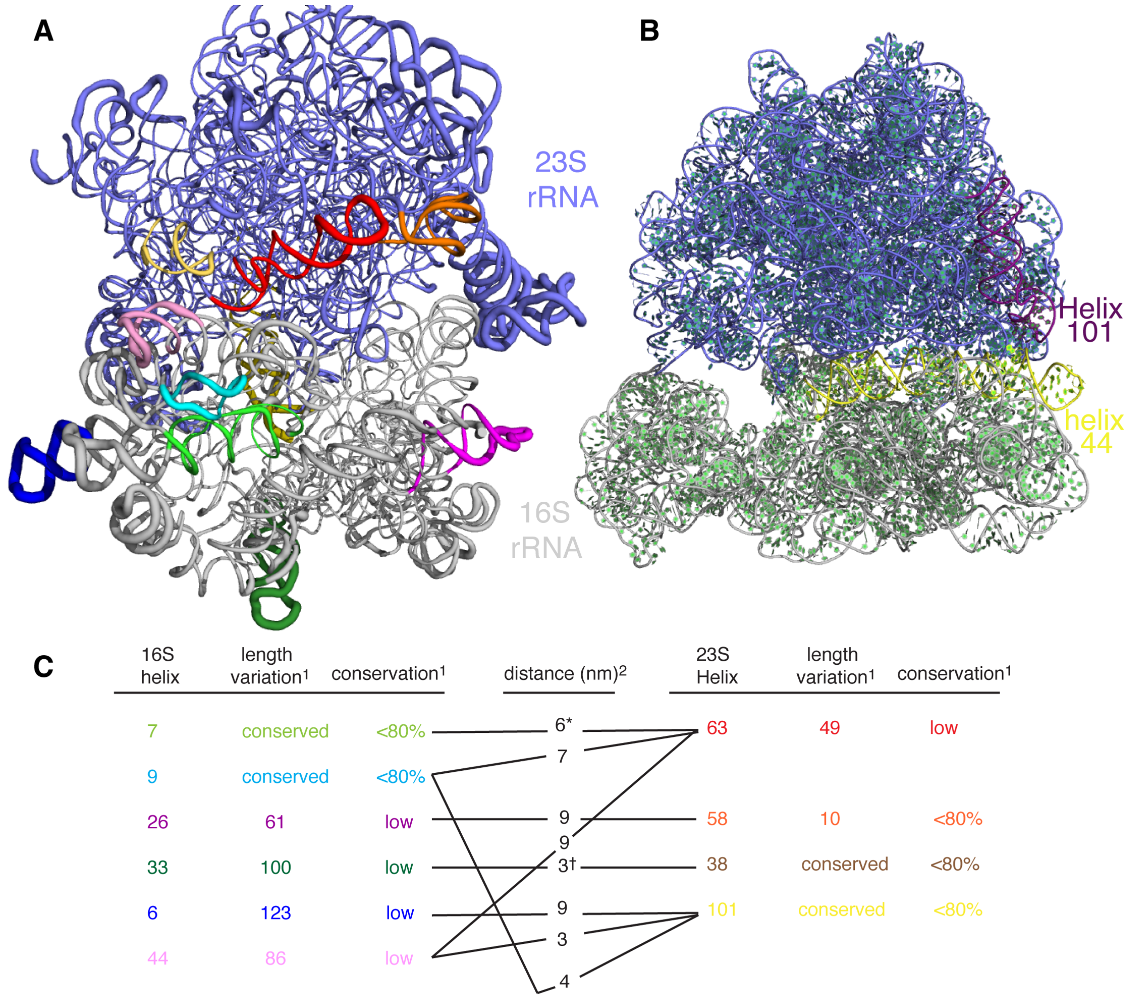

Figure S1. Stapling Sites In the Context of the Ribosome Structure. (**A**) Structural model of the *E. coli* ribosome highlighting (in colors) helices on the16S rRNA (cool colors) and on the 23S helices (warm colors) of low conservation that closely approach one another (<10 nm). (**B**) helix 44 and Helix 101 come into close contact and are well-removed from the tRNA binding sites (the hollow region on the left side). (**C**) Colors correspond to panel A. [1]Across bacteria, using *E. coli* as a reference. [2]From helix-end to helix-end, using the structure 5AFI. * Would require deleting off helices 8, 9, and 10. † Would involve tethering the head region to the central protuberance (both mobile regions).

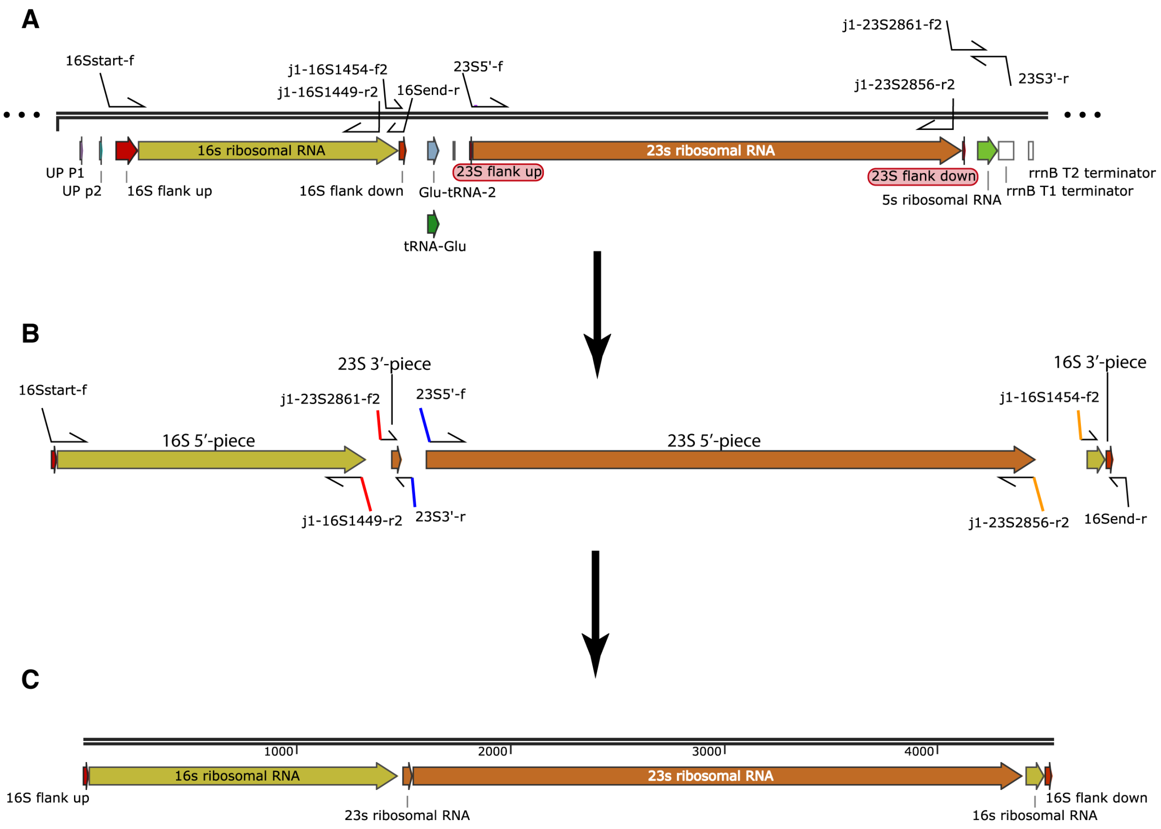

Figure S2. Strategy to synthesize ribo(h44H101). (**A**) A plasmid containing the rrnB operon is used as a template for three PCR reactions. One amplicon is short enough that the template can be omitted. (**B**) One fragment contains the majority of the 16S rRNA along with the leading flank (16S 5'-piece). A second small fragment corresponds to the final 85 nt of the 23S rRNA (23S 3'-piece). The third fragment is the majority of the 23S rRNA (23S 5'-piece). The fourth and final fragment corresponds to the final 89 nt of the 16S rRNA along with the trailing flank (16S 3'-piece). The primers were used to generate homology regions (red, blue, orange) for overlap extension PCR. The red and orange homology regions also install the double-stranded linker. The blue homology region contains a short GAGA insertion to close the stem of the original 5'/3'-termini of 23S with a tetraloop. (**C**) The final 4536 bp product following assembly and purification PCRs.

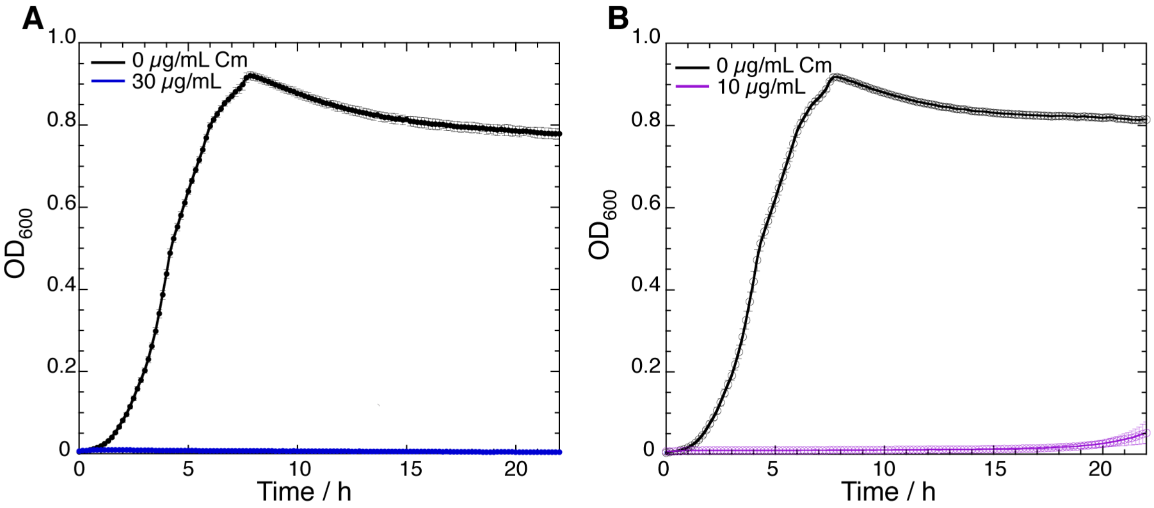

Figure S3. Negative Controls for O-ribo(h44H101) activity. (**A**) Growth curves of *E. coli* bearing pRSF- O-ribo(h44H101) and the p15A-O-cat reporter at 37˚C in liquid LB media *without* IPTG (see Table S2 for fit parameters). (**B**) Growth curves of *E. coli* bearing the p15A-O-cat reporter but *without* a plasmid encoding an orthogonal ribosome at 37˚C in liquid LB media (see Table S3 for fit parameters).

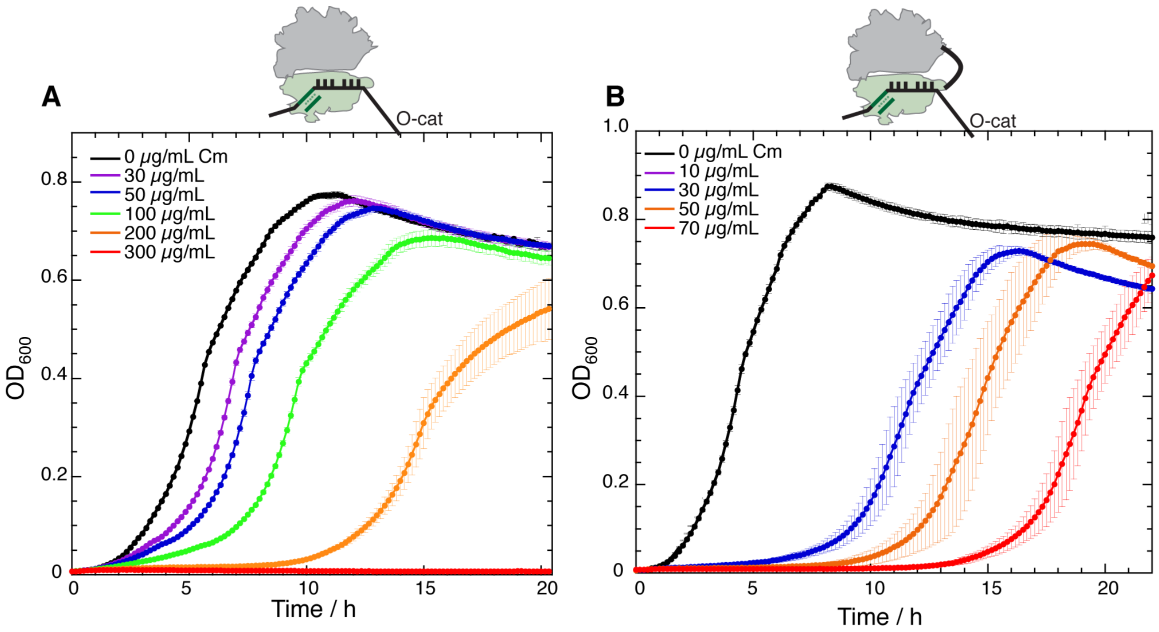

Figure S4. Positive Control for O-ribo(h44H101) activity. (**A**) Growth curves at 37˚C for cells transformed with pRSF O-ribo, an orthogonal ribosome with wild-type topology, and the p15A-O-cat reporter. The higher activity of this ribosome (ca. 4-fold relative to O-ribo(h44H101)) may be explained due to a higher copy number in the cell (see Figure 2A and B). (**B**) Expanded version of Figure 3C of the main text, showing growth curves at 37˚C for cells transformed with pRSF O-ribo(h44H101) and the p15A-O-cat reporter.

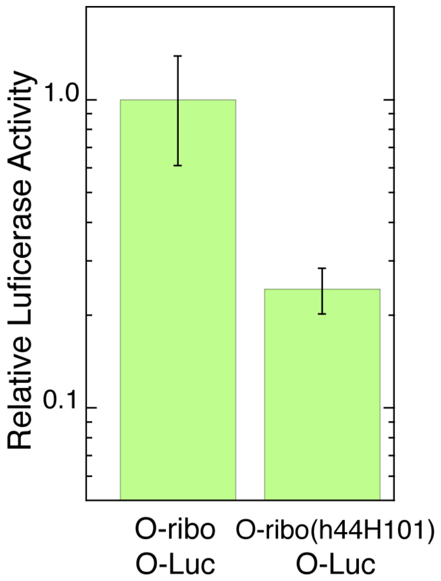

Figure S5. Luciferase activity generated by the translation of an orthogonal luciferase reporter (O-Luc) by O-ribo (an unstapled orthogonal ribosome) and O-ribo(h44H101). Bars represent means ± standard deviation from three biological replicates. Specific luciferase activity was assessed by subtracting the background levels of translation of O-Luc by wild-type (non-orthogonal) ribosome (about one order of magnitude down from the signal produced by these two orthogonal ribosomes).

III. Supplementary Tables

Table S1. Oligos used in this study

Name	Sequence (5' → 3')	Purpose
16Send-r	gagcactacaaagtacgcttctttaaggtaaggagg	Assembly of h44H101
16Sstart-f	gaagtttaattctttgagcgtcaaacttttaaattgaagag	Assembly of h44H101
23S3'-r	gcttagtcgcttaaccTCTCaaggttaagcctcacggttcattagtacc	Assembly of h44H101
23S5'-f	ggcttaaccttGAGAggttaagcgactaagcgtacacgg	Assembly of h44H101
j1-16S1449-r2	gcaCTGAACGGCTGTTGACCCCggttaagctacctacttcttttgcaacccactc	Assembly of h44H101
j1-16S1454-f2	gcgcaCTGACGGACATGGTCCTggagggcgcttaccactttgtg	Assembly of h44H101
j1-23S2856-r2	ccAGGACCATGTCCGTCAGtgcgcttacacacccggcctatc	Assembly of h44H101
j1-23S2861-f2	ccGGGGTCAACAGCCGTTCAGtgcgttgagctaaccggtactaatgaaccg	Assembly of h44H101
clone-pRSFbb-f	GAAGCGTACTTTGTAGTGCTCACACAGATTGTCTGATAGATGGCGGATGAGAGAAGATTTTCAGC	Clone h44H101 into pRSF
clone-pRSFbb-r	AAAAGTTTGACGCTCAAAGAATTAAACTTCGTAATGAATTACG	Clone h44H101 into pRSF
16S-ortho1-f	gggaacctgcggttggatcatgggattaCCTTAAAGAAGCGTAC	Site-directed mutagen. h44H101→ oh44H101
16S-ortho1-r	ctacggttaccttgttacgacttcacc	Site-directed mutagen. h44H101→ oh44H101
16S-ortho2-f	agatctggaggaataccgCAggcg	Site-directed mutagen. h44H101→ oh44H101
16S-ortho2-r	ctacgcatttcaccgctacacctgg	Site-directed mutagen. h44H101→ oh44H101
WS160f-G2252A	GTAGTTTGACTGGAGCGGTCTCCTCC	Site-directed mutagen.
WS160r-2252A	GGAGGAGACCGCTCCAGTCAAACTAC	Site-directed mutagen.
WS159f-G2553C	CAAGGGTATGGCTCTTCGCCATTTAAAG	Site-directed mutagen.
WS159r-G2553C	CTTTAAATGGCGAAGAGCCATACCCTTG	Site-directed mutagen.
oASD-probe	[biotin]TAATCCCATGATCCAACCGCAGGTTCCC	Northern blot

Table S2. *E. coli* transformed with pRSF- O-ribo(h44H101) and p15A- O-CAT, but no IPTG added to express ribosomes, scanning over Cm concentrations.

Cm / (μg mL^{-1})	Max rate / (OD h^{-1})	Lag time / h	Max OD
0	0.304 ± 0.005	2.55 ± 0.010	0.92 ± 0.010
30	0.042 ± 0.016	-0.197	0.012 ± 0.0005
50	0.024 ± 0.018	-1.177	0.012 ± 0.0007
70	0.020 ± 0.027	-1.47	0.011 ± 0.002
100	0.013 ± 0.020	57.35	0.012 ± 0.001

Table S3. *E. coli* transformed with p15A- O-CAT only, scanning over Cm concentrations.

Cm / (μg mL^{-1})	Max rate / (OD h^{-1})	Lag time / h	Max OD
0	0.311 ± 0.004	2.64 ± 0.067	0.922 ± 0.007
5	0.186 ± 0.008	12.25 ± 0.296	0.763 ± 0.024
10	0.040 ± 0.0190	13.66 ± 12.00	0.081 ± 0.044
20	0.065 ± 0.031	-0.11	0.011 ± 0.001
30	0.018 ± 0.027	-1.75	0.010 ± 0

Table S4. *E. coli* transformed with pRSF- O-ribo(h44H101) and p15A -O-CAT, induced with IPTG to express ribosomes, scanning over Cm concentrations.

Cm / (μg mL^{-1})	Max rate / (OD h^{-1})	Lag time / h	Max OD
0	0.279 ± 0.006	2.83 ± 0.053	0.877 ± 0.009
30	0.188 ± 0.007	9.48 ± 0.489	0.738 ± 0.009
50	0.212 ± 0.004	12.68 ± 0.931	0.761 ± 0.0175
70	0.204 ± 0.005	17.09 ± 0.585	0.709 ± 0.025
100	0.031± 0.032	13.15 ± 11.47	0.039 ± 0.011

Table S5. *E. coli* transformed with pRSF- O-ribo and p15A- O-CAT, induced with IPTG to express ribosomes, scanning over Cm concentrations.

Cm / (μg mL^{-1})	Max rate / (OD h^{-1})	Lag time / h	Max OD
0	0.213 ± 0.002	3.66 ± 0.015	0.774 ± 0.006
30	0.241 ± 0.006	5.19 ± 0.033	0.762 ± 0.011
50	0.227 ± 0.003	5.89 ± 0.018	0.747 ± 0.008
100	0.207 ± 0.003	7.77 ± 0.019	0.686 ± 0.017
200	0.139 ± 0.019	12.77 ± 0.117	0.542 ± 0.062
300	0.003 ± 0.0003	-2.34	0.010 ± 0.0003
400	0.004 ± 0.0005	-1.84	0.010 ± 0.0004

Table S6. *E. coli* transformed with pRSF-O-ribo((h44H101)G2252A) and p15A-O-CAT, induced with IPTG to express ribosomes, scanning over Cm concentrations.

Cm / (μg mL^{-1})	Max rate / (OD h^{-1})	Lag time / h	Max OD
0	0.234 ± 0.005	4.27 ± 0.060	0.781 ± 0.003
10	0.183 ± 0.007	9.51 ± 0.189	0.706 ± 0.012
20	0.198 ± 0.011	16.28 ± 0.498	0.687 ± 0.033
30	0.055 ± 0.009	14.59 ± 14.57	0.132 ± 0.213
50	0.003 ± 0.002	-1.54	0.006 ± 0.0008

Table S7. *E. coli* transformed with pRSF-O-ribo((h44H101)G2553C) and p15A-O-CAT, induced with IPTG to express ribosomes, scanning over Cm concentrations.

Cm / (μg mL^{-1})	Max rate / (OD h^{-1})	Lag time / h	Max OD
0	0.233 ± 0.005	4.53 ± 0.063	0.783 ± 0.006
10	0.186 ± 0.003	9.39 ± 0.228	0.716 ± 0.006
20	0.205 ± 0.010	15.50 ± 0.086	0.712 ± 0.017
30	0.024 ± 0.003	22.97 ± 0.803	0.055 ± 0.058
50	0.002 ± 0.001	-1.99	0.005 ± 0.0008

Table S8. *E. coli* transformed with pRSF- O-ribo((h44H101)G2252A), scanning over IPTG concentrations.

IPTG / mM	Max rate / (OD h^{-1})	Lag time / h	Max OD
0	0.331 ± 0.015	2.77 ± 0.064	0.926 ± 0.006
0.01	0.324 ± 0.015	2.74 ± 0.067	0.911 ± 0.009
0.1	0.259 ± 0.004	2.82 ± 0.080	0.863 ± 0.006
1.0	0.246 ± 0.008	3.15 ± 0.089	0.841 ± 0.009

Table S9. *E. coli* transformed with pRSF- O-ribo((h44H101)G2553C), scanning over IPTG concentrations.

IPTG / mM	Max rate / (OD h^{-1})	Lag time / h	Max OD
0	0.328 ± 0.007	2.78 ± 0.057	0.911 ± 0.005
0.01	0.322 ± 0.006	2.76 ± 0.063	0.913 ± 0.004
0.1	0.248 ± 0.009	2.86 ± 0.075	0.851 ± 0.004
1.0	0.228 ± 0.005	3.22 ± 0.047	0.824 ± 0.006

Table S10. *E. coli* transformed with pRSF- O-ribo(h44H101), scanning over IPTG concentrations.

IPTG / mM	Max rate / (OD h^{-1})	Lag time / h	Max OD
0	0.317 ± 0.007	2.71 ± 0.066	0.919 ± 0.0045
0.01	0.314 ± 0.004	2.71 ± 0.059	0.912 ± 0.001
0.1	0.250 ± 0.006	2.80 ± 0.059	0.854 ± 0.007
1.0	0.238 ± 0.003	3.16 ± 0.037	0.825 ± 0.005

IV. Supplementary References

[1] K. Wang, A. Sachdeva, D. J. Cox, N. W. Wilf, K. Lang, S. Wallace, R. A. Mehl, J. W. Chin, *Nature Chemistry* **2014**, *6*, 393–403.

[2] K. Wang, H. Neumann, S. Y. Peak-Chew, J. W. Chin, *Nature Biotechnol.* **2007**, *25*, 770–777.

[3] J. C. Alwine, D. J. Kemp, G. R. Stark, *Proc. Natl. Acad. Sci. USA* **1977**, *74*, 5350–5354.
